# Supplementary material for: Novel Hydrogen Sulfide Hybrid Derivatives of Keap1-Nrf2 Protein–Protein Interaction Inhibitor Alleviate Inflammation and Oxidative Stress in Acute Experimental Colitis
Source: Antioxidants (Basel). 2023 May 8;12(5):1062. doi: 10.3390/antiox12051062 (PMC10215391; doi:10.3390/antiox12051062)

antioxidants

Supporting information

Novel Hydrogen Sulfide Hybrid Derivatives of Keap1-Nrf2 Protein–Protein Interaction Inhibitor Alleviate Inflammation and Oxidative Stress in Acute Experimental Colitis

Xian Zhang [1,2,†], Keni Cui [1,2,†], Xiaolu Wang [1], Yuanyuan Tong [1,2], Chihong Liu [1,2], Yuechao Zhu [1,2], Qidong You [1,2,*], Zhengyu Jiang [1,2,*] and Xiaoke Guo [1,2,*]

[1] State Key Laboratory of Natural Medicines and Jiang Su Key Laboratory of Drug Design and Optimization, China Pharmaceutical University, Nanjing 210009, China
[2] Department of Medicinal Chemistry, School of Pharmacy, China Pharmaceutical University, Nanjing 210009, China
* Correspondence: youqd@cpu.edu.cn (Q.Y.); jzy@cpu.edu.cn (Z.J.); 1020142412@cpu.edu.cn (X.G.)
† These authors contributed equally to this work.

Figure S1. DDO-1901 in PBS (1 % DMSO) with esterase 20 unit/mL at 37°C

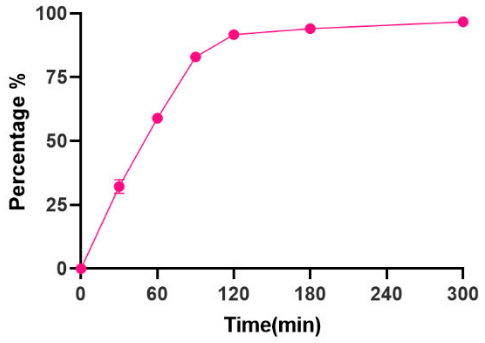

Figure S2. DSS-induced cell damage at different concentrations (from 2.5 mg/mL to 40 mg/mL). Cell viability was measured using the MTT assay

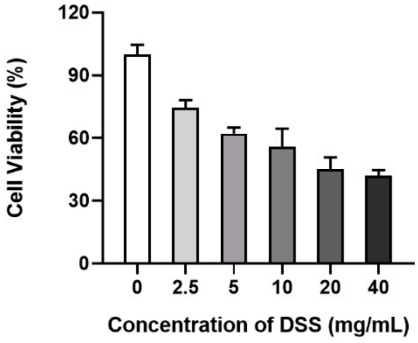

Supplement: Supplementary file 1 [file antioxidants-12-01062-s001.zip › antioxidants-2261601-supplementary.pdf]
